# Supplementary material for: Characterizing neural coding performance for populations of sensory neurons: comparing a weighted spike distance metrics to other analytical methods
Source: Front Neurosci. 2023 Jun 5;17:1175629. doi: 10.3389/fnins.2023.1175629 (PMC10277732; doi:10.3389/fnins.2023.1175629)
Supplement: Supplementary file 1 [file Data_Sheet_1.docx]

## Supplementary Information


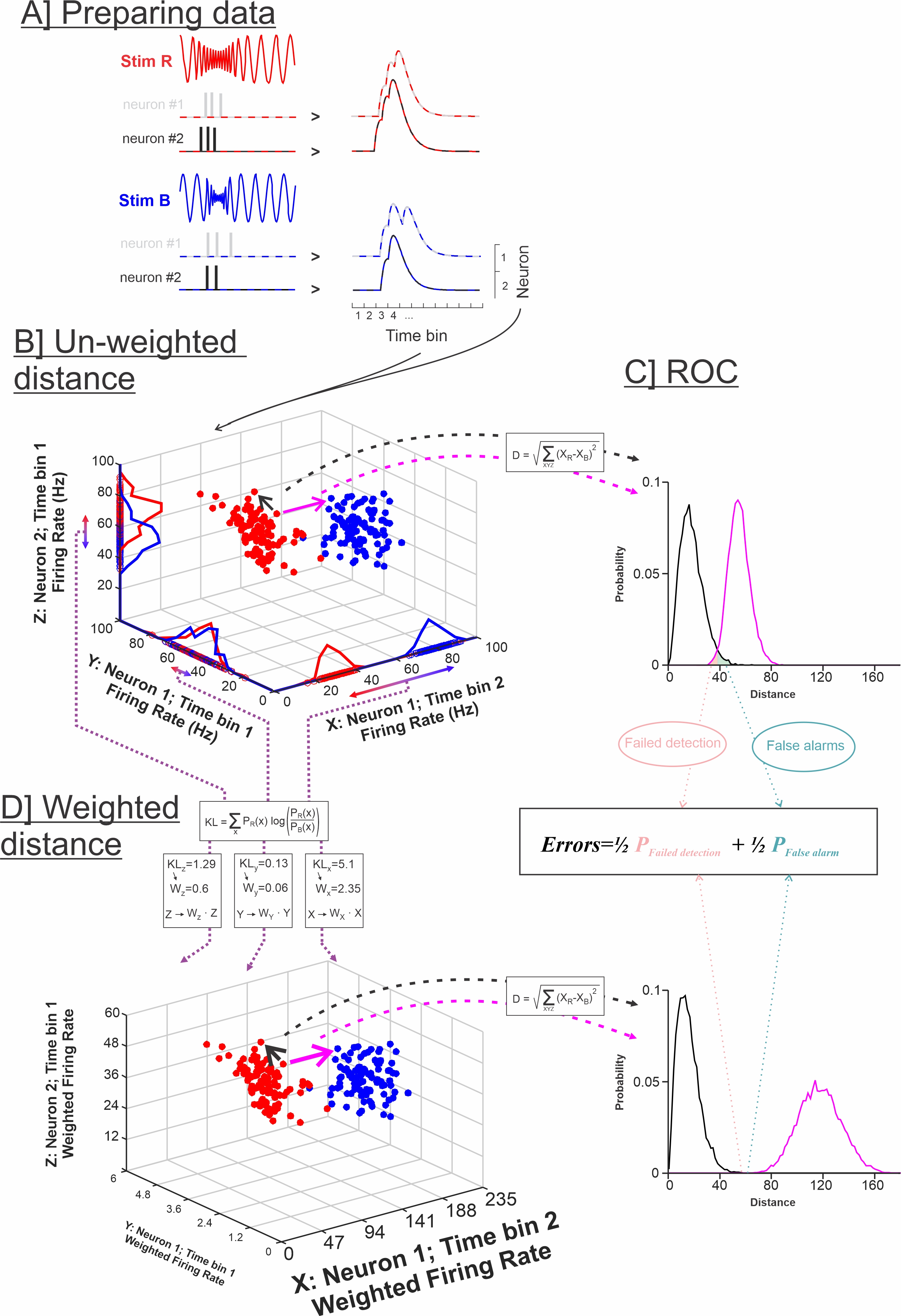


**Supplementary Information Figure 1:** Weighted and unweighted Euclidean distance analysis. **A.** Spike train responses to two different stimuli are prepared as for other analyses and replicates the procedure used in van Rossum (2001). Binary spike trains are convolved with an alpha function (resembling the time course of post-synaptic potentials) resulting in an estimate of instantaneous firing frequency. The firing rate for each neuron and at each time point can then be used as inputs to calculate the spike-train metric distance. Each population response is taken as a data point in multi-dimensional Euclidian space with each orthogonal dimension representing the firing rate of one neuron at one time point. Therefore the number of dimensions is the product of [number of time points] x [number of neurons]. We depict 3 dimensions here for illustration purposes. A large random subset of pairs of responses (to the two stimuli – red and blue) is compared by calculating their Euclidean distance (magenta). Considering the number of stimuli pairs and the number of repeats, there are often millions of possible pair-wise comparisons, therefore, requiring a random sub-sample of combinations to be taken. Pairs of responses to the same stimulus are also compared (black Euclidean distance) so we can compare the inter-stimuli to the intra-stimulus distances. The inter- and intra-stimulus distances are expressed as distributions (magenta and black distributions respectively). The overlap between the distributions can be quantified, assuming an ideal observer, using a ROC analysis to obtain a minimum error probability. D. When proceeding to weigh each dimension in the Euclidean distance analysis, the overlap in the distribution in each dimension is quantified. The distributions in each of the 3 dimensions displayed in B are displayed along the analysis and we can see that some overlap almost completely (e.g. in dimension Y) whereas some do not overlap at all, (in dimension X) leading to small and large Kullback-Leibler divergences respectively (see equation and numbers in the boxes). The weights are the result of a normalization (dividing by the average KL divergence across all dimensions) of the KL values so that the average weight is 1 for each pair-wise comparison. Euclidean distance is calculated after multiplying the firing rate in each dimension by the corresponding weight (notice the change in axis scale between the 3D plots in B and D; font size for axis labels in D reflect the change in weight). The overlap between the distributions for intra- and inter-stimuli Euclidean distances is thereby reduced (see bottom-right plot used for ROC analysis) and the discrimination error probability is minimized.


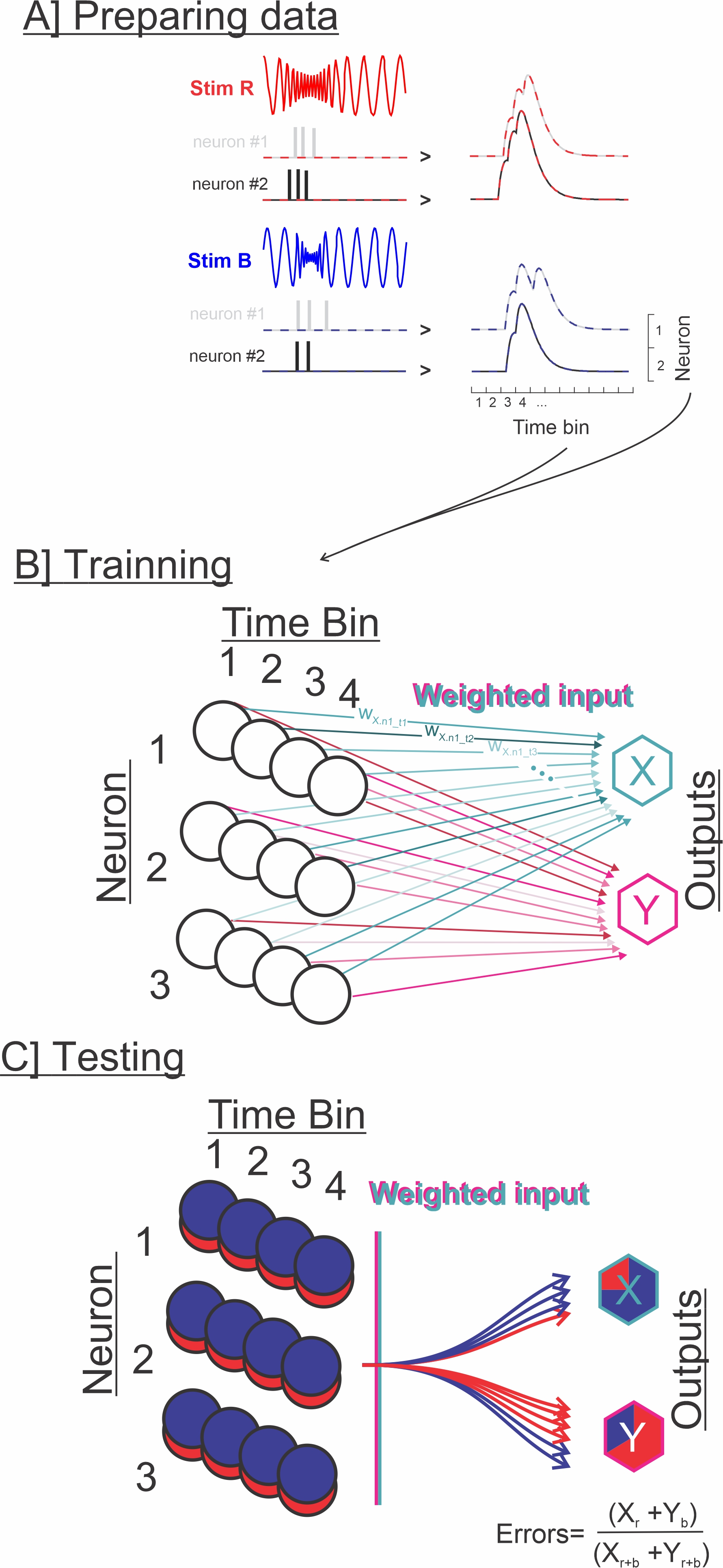
**Supplementary Information Figure 2**: Using a SOM neural network to test responses discriminability. **A.** Spike train responses to two different stimuli are prepared the same way it is in the other analysis used in this paper: binary spike trains are convolved with an alpha function (resembling the time course of post-synaptic potentials) resulting in an estimate of instantaneous firing frequency. The firing rate for each neuron and at each time point can then be used as inputs to the neural net **B.** The SOM neural network available in Matlab 2017b (Mathworks, Natick, MA) is a shallow unsupervised neural network with an input layer and an output layer. The weights between the input and output nodes are optimized based on the variability of the input patterns to maximize the clustering in separate groups. We use networks with the same number of inputs as dimensions in our population responses (the product of [number of time points] x [number of neurons]). The number of outputs is equal to the number of stimuli; here we tested pair-wise discrimination so we have 2 output nodes. **C.** After training, a different subset of data is used for testing. Classification accuracy is calculated, where an error probability of 0 is reached if all the responses to the two stimuli as classified in separate outputs and 0.5 error probability indicating that each output receives data point from either stimulus equally (chance level).


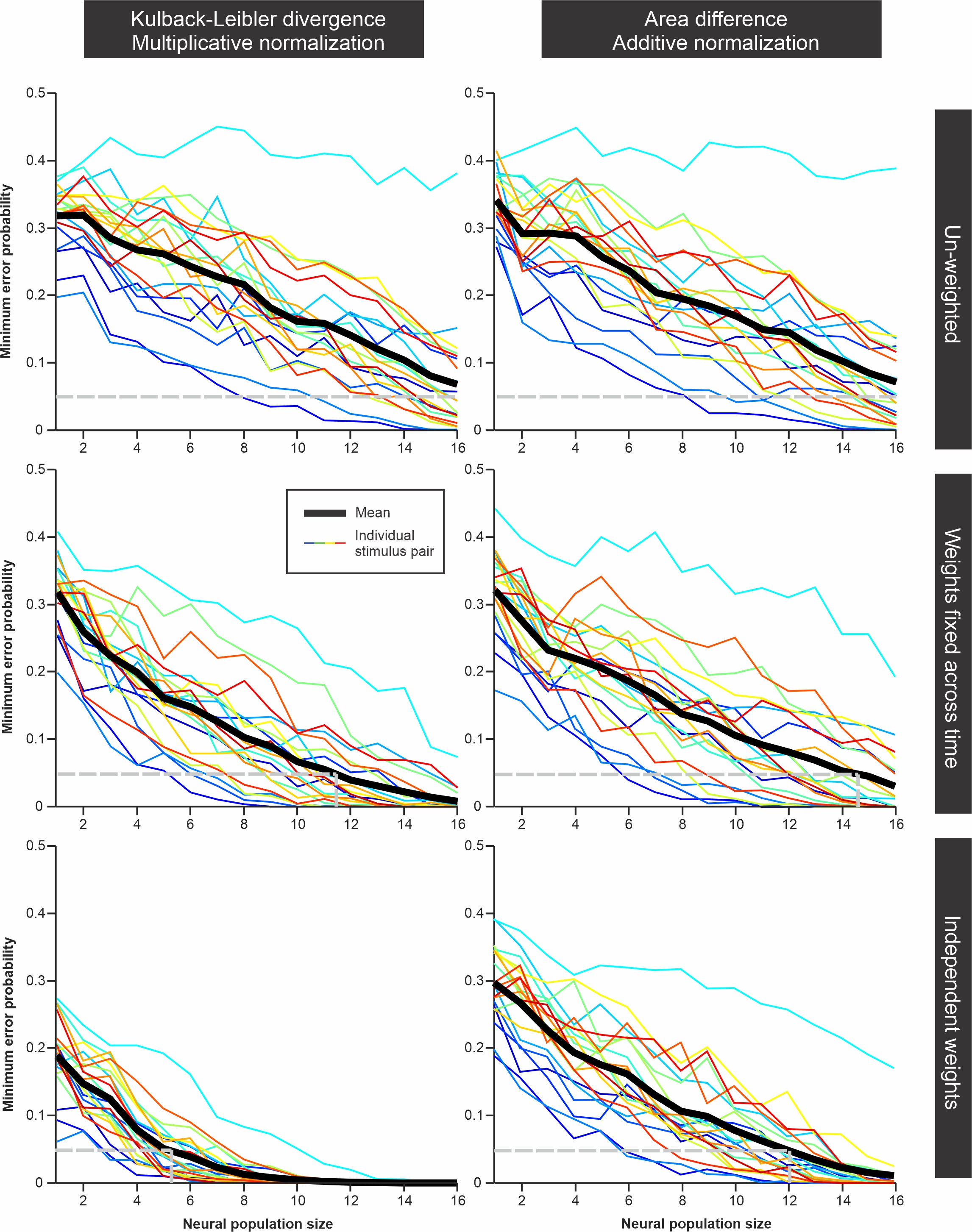


**Supplementary Information Figure 3:** Comparison of the WED discrimination analysis using two different weighting methods. In the plots in the **left column**, we calculate the Kullback Liebler divergence for the distributions of firing rates in each dimension for a given stimulus pair (see Supp Fig. 2). These KL-based distances are then normalized by dividing with the mean KL-based distances across dimensions thereby giving an average weight of 1 in each comparison. In the **right column**, we quantify the overlap between distributions by taking the absolute value of the differences between the two distribution curves (this value will vary between 0 and 2 since each distribution has an integral of 1). The mean -across dimensions- of these overlap measurements is then shifted to 1 (by adding 1-mean) and any negative values are set to 0. The two weighting methods are fully implemented in the two **bottom plots**. In the **middle plots,** the weights calculated independently for each dimension are averaged across time points for each neuron in the population before calculating the Euclidean distance (i.e. one neuron=one weight). In the top plots the different dimensions are not weighted (all weights =1) thus the two plots differ only slightly reflecting the difference in the randomly selected pairs of stimuli/repeats used in the analysis. The plots displayed here are based on the olfactory responses to 7 odors and each of the 21 pair-wise comparisons is shown in a different color with the average across stimuli pairs in black. We highlight with a grey dashed line the 0.05 error probability, and the average number of neurons required to reach this reliability level, as a simple way to compare discrimination accuracy across datasets/analysis. It is interesting to note that the KL-based analysis outperforms the area-based method, particularly when weight can vary in time (bottom two plots). We have observed, however, that for some datasets (data not shown), the more pronounced weighting of our KL-based method led to results that were maybe unrealistically good. Due to the multiplicative normalization, this method can attribute very strong weights to just a few time points/neurons with the other weights being much weaker. It is unclear whether biological systems could implement such extreme weighting, particularly across time. More biologically realistic estimates could therefore be based on the method presented on the right (where weights are normalized by an additive process and thus vary less strongly across dimensions) and/or by keeping weights constant across time.


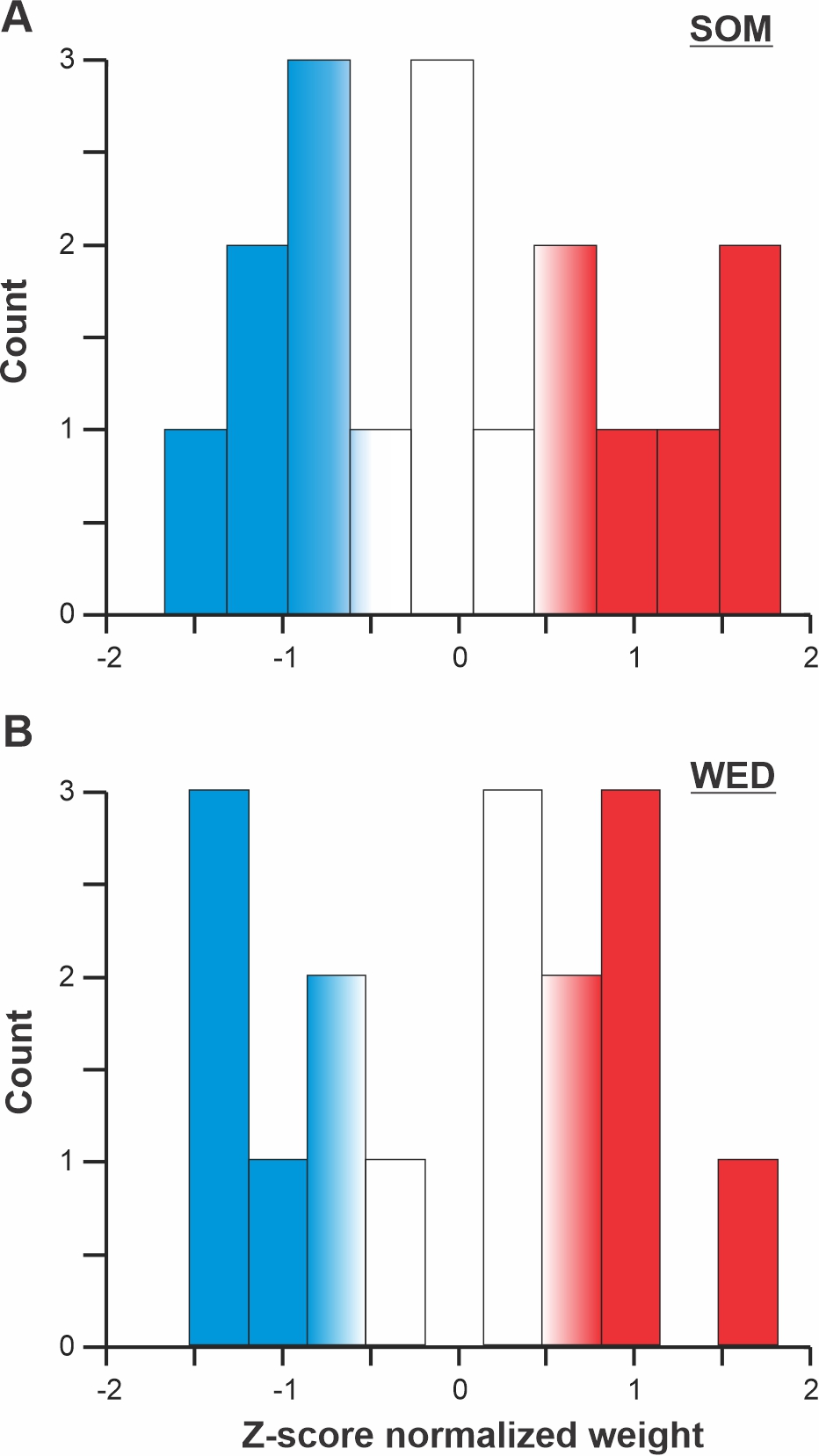


**Supplementary Information Figure 4:** Distribution of weights for each of the 16 neurons in one analysis of discrimination for a pair of odor stimuli. **A.** Weights extracted from the trained SOM neural net performing the discrimination task. **B.** Weights for the WED discrimination analysis of the same pair of stimuli. The weights are z-score normalized for comparison. We see that efficient discrimination required ~ 6 neurons to be strongly weighted (highlighted in red; 8-9 neurons were weighted more than the average). This secondary analysis could be useful as a prediction of the connectivity patterns that could present in the biological system (e.g. how many neurons converged onto a single decoding postsynaptic cell).


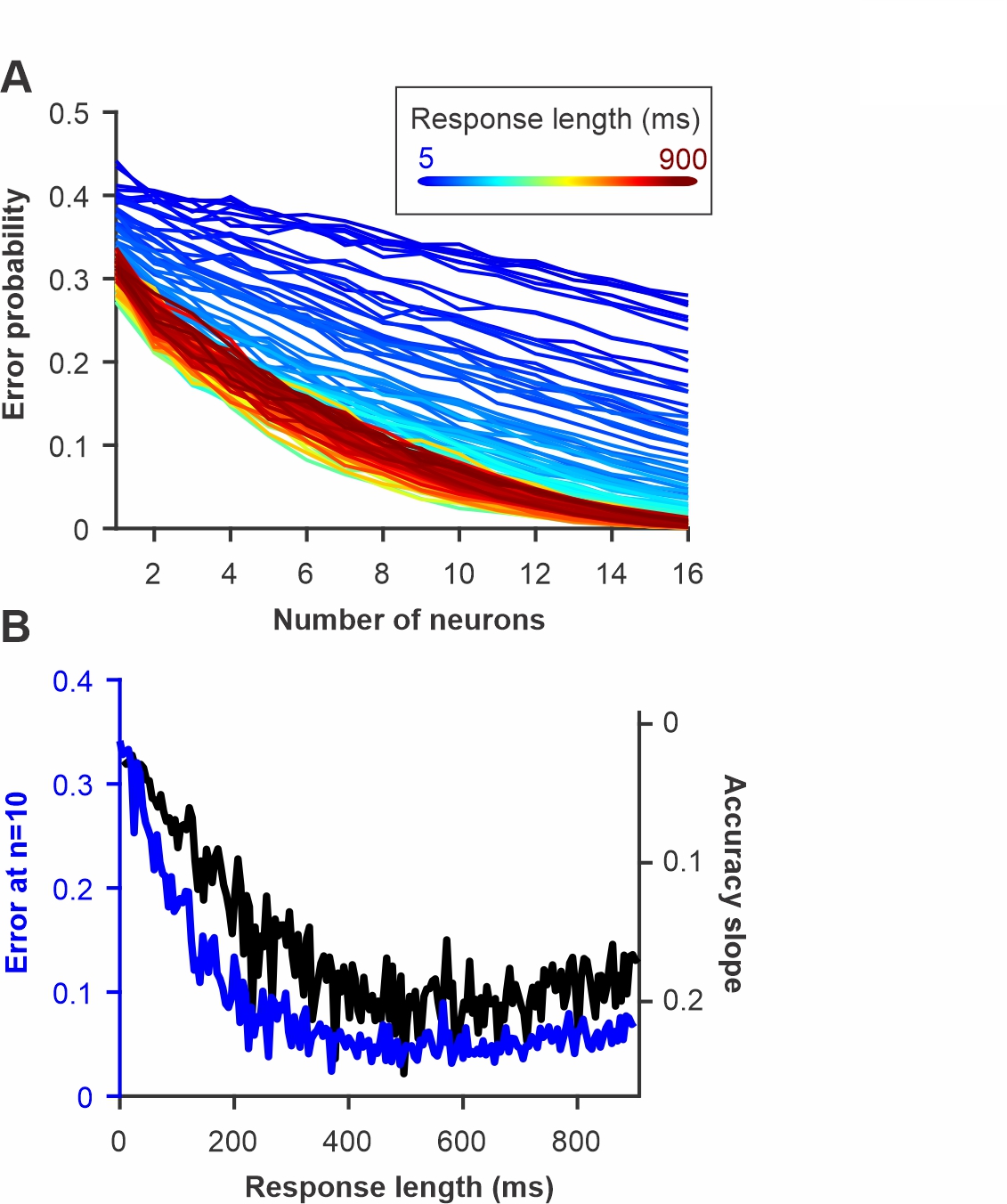


**Supplementary Information Figure 5:** Discrimination accuracy for pairs of olfactory stimuli as increasing lengths of the neural responses after stimulus onset are included in the analysis. **A.** Error probability as a function of number of neurons included in the analysis and duration of the response. **B.** Discrimination performance quantified by the error rate for a population of 10 neurons (blue) or as the slope of the curves in A (see Fig. 6). We see that discrimination accuracy should increase quickly in the first 200 ms of the response but then plateaus. These estimates can be related to the behavioral performance of the animal. For behavioral response that happen quickly after stimulus onset, neural responses pattern should contain enough information in the initial portion of their responses to support accurate discrimination. Interestingly, it has been shown that moths can perform accurate behavioral discrimination within a few hundred ms of the odor presentation for these type of stimuli.


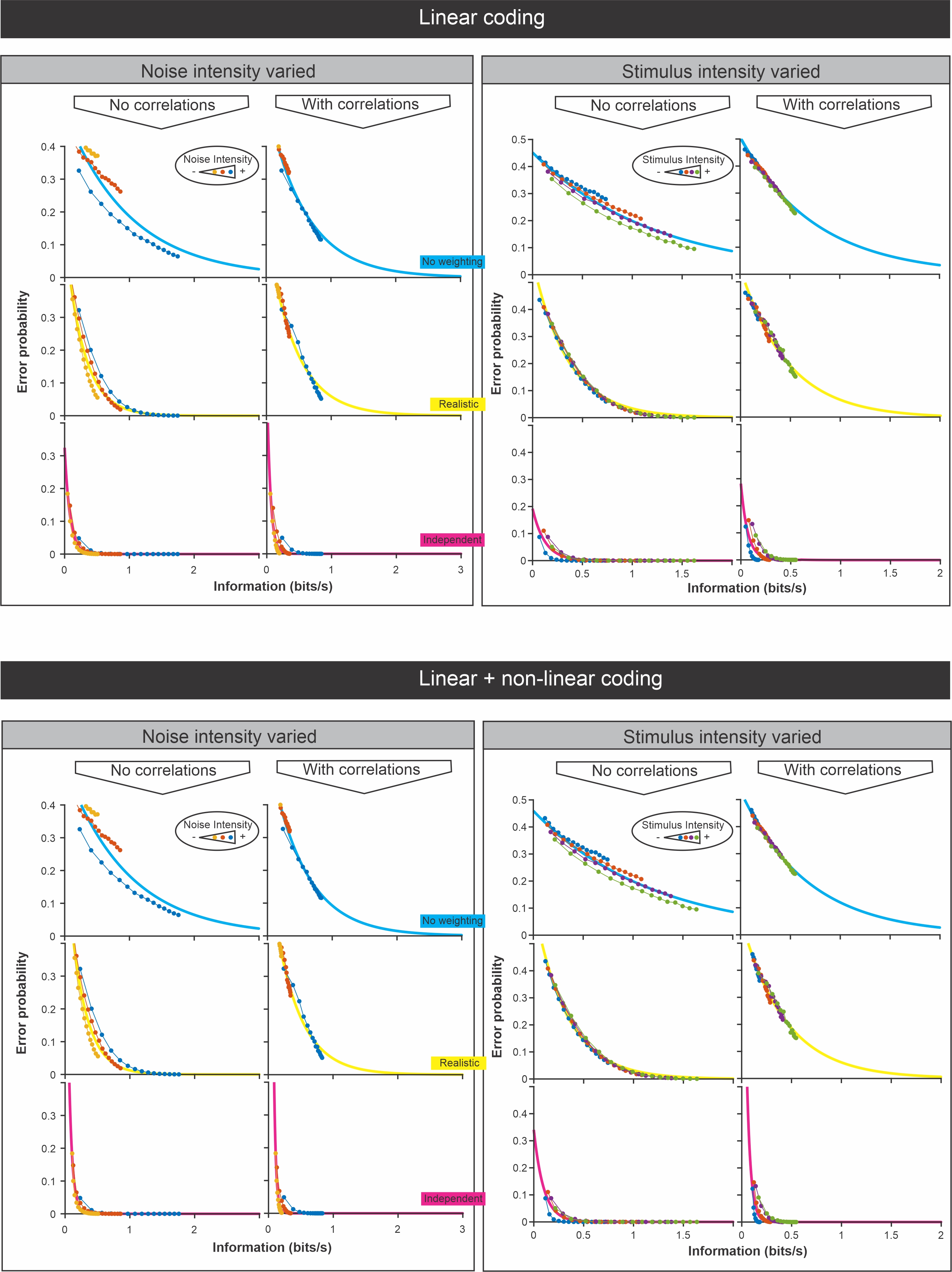


**Supplementary Information Figure 6:** Comparison of discrimination error probability and information content of LIF model responses to frozen white noise stimuli. This figure complements Fig. 7 of the main text. We performed the analysis using response-stimulus coherence to estimate information, which give a lower-bound estimate of the information content because it considers only the information encoded linearly. We also performed the analysis using response-response coherence which provides an upper-bound estimate of information content that includes both linear and non-linear coding (see Borst and Theunissen 1999 for more details). Our LIF model was adjusted to be in a linear regime therefor there is no difference between the two sets of plots (upper half vs bottom half of the figure). To change the signal to noise ratio we changed the amount of noise while keeping the strength of the signal constant (left half of this figure; similar to Fig. 7 although here the overall signal strength was lower than in Fig. 7). Alternatively, we changed the stimulus strength keeping the noise constant (right half of this figure). The analysis was performed with noise correlation present of not (see Fig. 8 for more details). Each panel presents the analysis with the three different weighting methods (no weighting in blue, realistic weighting in yellow and optimized weighting in magenta) that are used in Fig. 6-8. We fitted exponential curves in each plot to show the high correlation between information content and discrimination accuracy. We note that this relationship changes with the discrimination decoding methods as some methods can leverage better the most informative parts of the responses and thus lead to better discrimination although the information content doesn’t change. The relationship can also be affected but the characteristics of the stimulus as shown by the fact that results for different signal-to noise ratios follow slightly different curves in some plots (compare for example the 3 curves in the “realistic” analysis when the noise intensity is varied and no noise correlations are present).
